# Supplementary material for: Deregulated Transcriptome as a Platform for Adrenal Huntington’s Disease-Related Pathology
Source: Int J Mol Sci. 2024 Feb 11;25(4):2176. doi: 10.3390/ijms25042176 (PMC10888552; doi:10.3390/ijms25042176)
Supplement: Supplementary file 1 [file ijms-25-02176-s001.zip › ijms-2835922-supplementary.pdf]

Deregulated Transcriptome as a Platform for Adrenal Huntington's Disease-Related Pathology

Anna Olechnowicz [1,2], Małgorzata Blatkiewicz [1], Karol Jopek [1], Mark Isalan [3,4], Michal Mielcarek [3,4] and Marcin Rucinski [1,*]

[1] Department of Histology and Embryology, Poznan University of Medical Sciences, 61-701 Poznan, Poland; anna.olechnowicz@student.ump.edu.pl (A.O.); mblatkiewicz@ump.edu.pl (M.B.); karoljopek@ump.edu.pl (K.J.)

[2] Doctoral School, Poznan University of Medical Sciences, 60-812 Poznan, Poland

[3] Department of Life Sciences, Imperial College London, Exhibition Road, London SW7 2AZ, UK; m.isalan@imperial.ac.uk (M.I.); mielcarekml@gmail.com (M.M.)

[4] Imperial College Centre for Synthetic Biology, Imperial College London, London SW7 2AZ, UK

* Correspondence: marcinruc@ump.edu.pl

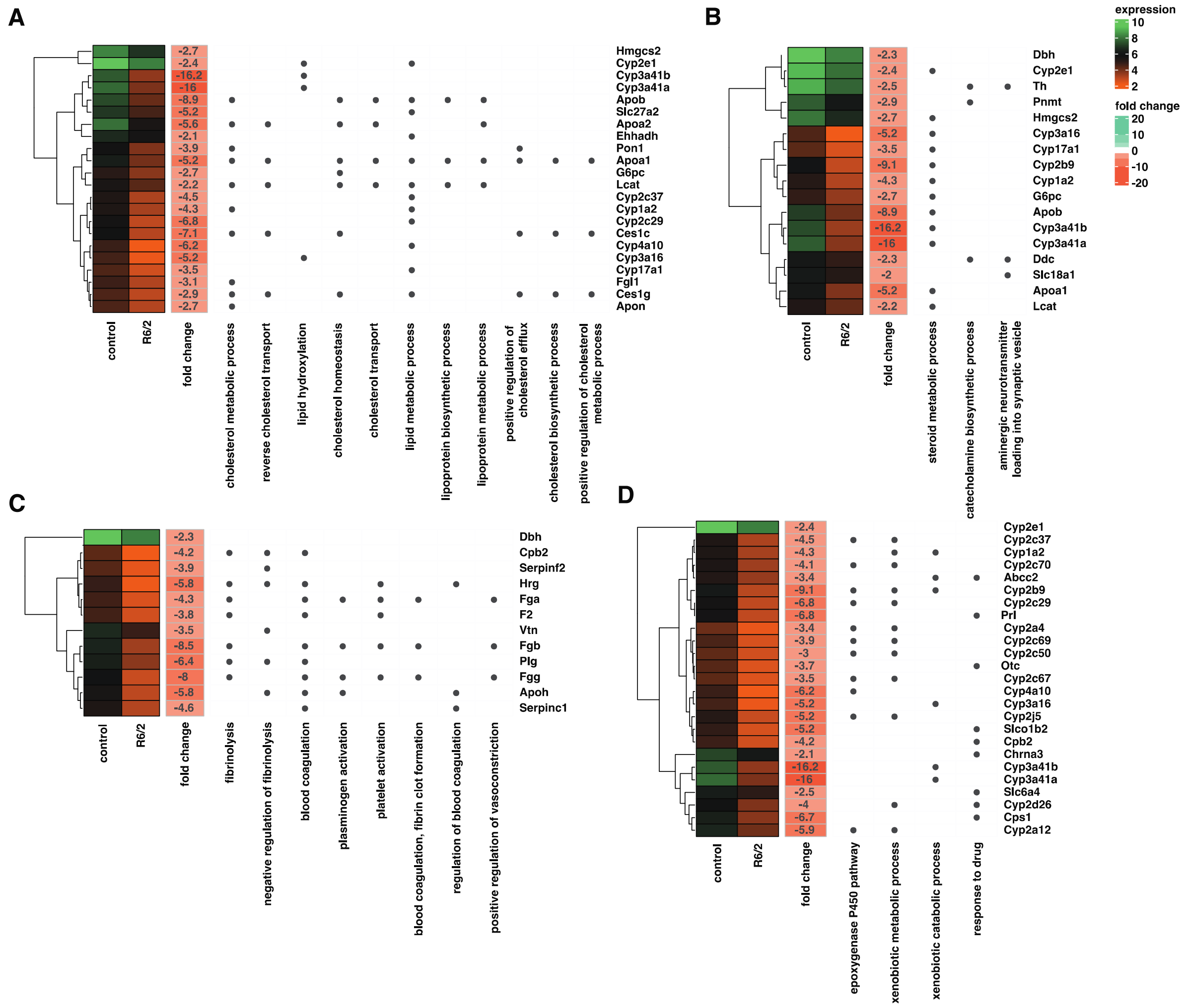

Supplementary Figure S1. Heatmap of differently expressed genes in the R6/2 group compared to the genetic background control assigned to adequate gene sets in the cluster of cholesterol metabolism (A), steroids and catecholamines metabolism (B), blood coagulation (C), and xenobiotic metabolism (D) processes. Each group and gene set are color-coded. Two left columns show the mean expression of each gene with a color gradient from green (highest expression) to red (lowest expression). The middle column represents the fold change of the gene expression between the R6/2 group and the

genetic background control group from turquoise (highest positive fold change) to red (highest negative fold change). The central part of the heat map shows the gene belonging to the gene sets by using black dots.

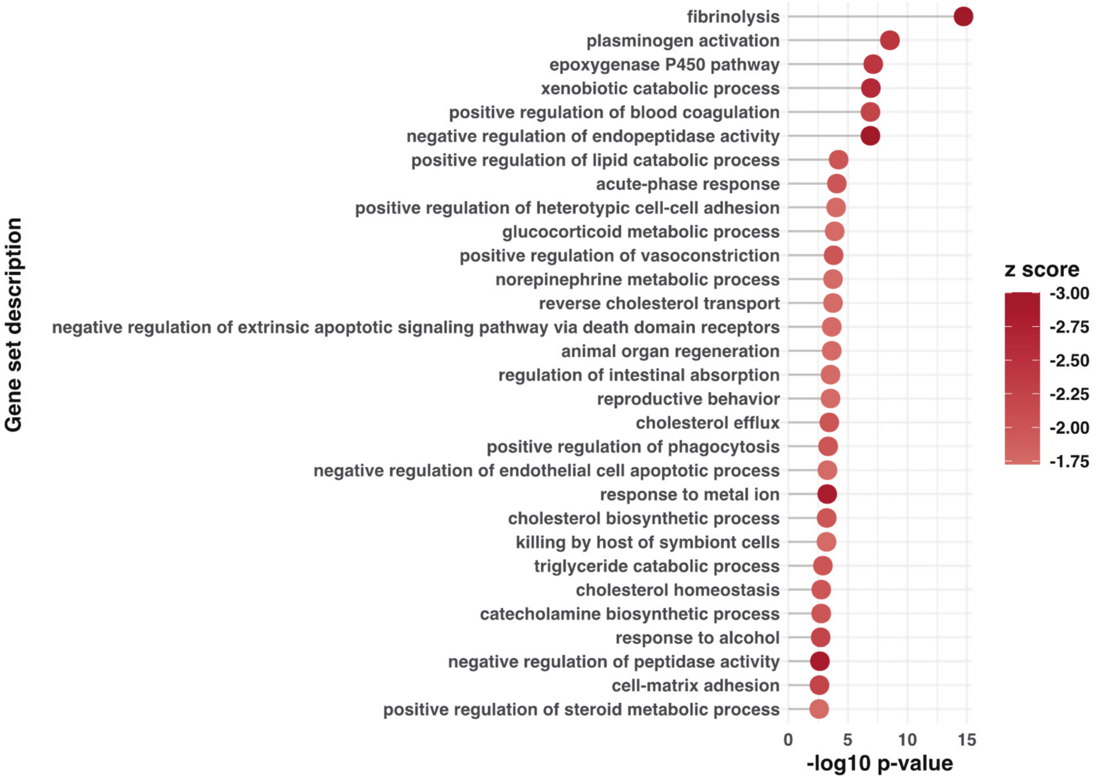

Supplementary Figure S2. Enrichment analysis of ontological terms using the Genetonic library. Dot plot representing down-regulated processes in the adrenal from the R6/2 group compared to the genetic background control. Various shades of brown indicate fold change.
